# Effect of Tartaric Acid on the Printable, Rheological and Mechanical Properties of 3D Printing Sulphoaluminate Cement Paste

**DOI:** 10.3390/ma11122417

**Published:** 2018-11-29

**Authors:** Mingxu Chen, Xiangyang Guo, Yan Zheng, Laibo Li, Zhen Yan, Piqi Zhao, Lingchao Lu, Xin Cheng

**Affiliations:** 1 School of Materials Science and Engineering, University of Jinan, Jinan 250022, China; 15806612606@163.com (M.C.); Zhengyan_2017@126.com (Y.Z.); Laibo_li@163.com (L.L.); yanzhen_ujn@163.com (Z.Y.); 2 Shandong Provincial Key Lab. of Preparation and Measurement of Building Materials, University of Jinan, Jinan 250022, China; mse_zhaopq@ujn.edu.cn; 3 Shandong Provincial Academy of Building Research, Jinan 250022, China; gxy0818@163.com

**Keywords:** 3D printing, rheological properties, tartaric acid, sulphoaluminate cement, thixotropy

## Abstract

Rapid setting and low viscosity of sulphoaluminate cement (SAC) make it difficult to be extruded by 3D printing (3DP) technique. In this study, the effect of tartaric acid (TA) on printability, rheology and mechanical property of 3DP SAC paste is investigated. The experimental results indicate that the setting time, hydration evolution and apparent viscosity of SAC paste can be well controlled by adding a proper amount of TA to satisfy the requirements of 3DP. An excellent structure of SAC paste with the ultimate deformation rate less than 10% can be printed without compromising mechanical strength.

## 1. Introduction

3D printing (3DP) is a kind of rapid prototyping technology based on digital model, which constructs the objects layer by layer [1,2]. Due to many advantages of 3DP, including fast production, cost effectiveness, and shape diversity, it has been widely used in architecture, medicine, aviation, and space [3,4]. In general, 3DP mainly consists of two types: extrusion-based printing and powder-based printing. Compared with powder-based 3DP, extrusion-based 3DP is more effective to fabricate the final products without removing the unprinted materials. However, no matter which type of 3DP is used, the printing materials should have a good adhesive property to ensure a strong interfacial bonding between each layer and a high viscosity to guarantee the shape stability after 3DP [5,6].

However, the application of 3D printing building materials is recent [7]. The printing model is designed based on the computer, and cementitious material is extruded from 3D printer through a technology similar to fused deposition modeling (FDM) [8,9]. Compared with the traditional casting method, 3DP method is independent of molds and cutting technologies, which are expensive and labor consuming to generate the desired shapes [10,11]. The products with diverse shapes can be directly printed from the 3D printer when the properties of cementitious materials satisfy the requirements of 3DP, leading to a significantly reduction in construction time and fabrication cost [12,13]. 3DP is also very easy to print complex and curvilinear buildings that are difficult to construct in other ways [14].

Many research works have been conducted to investigate the structures and properties of 3DP cementitious materials. Shakor et al. investigated the porosity and mechanical strength of calcium aluminate cement paste by powder-based 3DP technique [15]. Ma et al. revealed the effect of copper tailings on the printability and mechanical properties of cementitious composite by 3DP, and indicated that 30.0 wt.% addition of waste copper tailings greatly improved the mechanical strength [16]. Ketel et al. proposed a printability index to ensure the geometric precision for 3DP products by measuring the variation of the rheology [17]. Soltan et al. developed a large-scale self-reinforced 3DP cementitious composite, and found that the hybrid additions of composites or substitutions and water significantly affect the mechanical performance of the corresponding products [18]. Besides, in the field of 3D concrete printing (3DCP), Buswell et al. offered a roadmap to inspire and guide the future research effort [19]. Reiter et al. gave a review of structural build up in 3DP concrete [20].

SAC is a kind of rapid-hardening cement, exhibiting two desirable features for 3DP, i.e., high early strength and fast hardening [21]. These two features are beneficial to improve the early strength and shorten the production period of 3DP. However, cementitious materials and its mix proportion are very difficult to improve the buildability and printability. Therefore, admixtures are indispensable for 3DP cement paste to handle these behaviors [22].

In this study, the effect of tartaric acid (TA) on the printable, rheological and mechanical properties of SAC paste is investigated by extrusion-based 3DP. Firstly, the setting time of SAC will be adjusted by adding TA to satisfy the printing requirement. Secondly, based on the previous works, rheology of cementitious materials is a key factor [23,24,25] to determine whether it can be successfully printed or not. Meanwhile, rheology is closely related to the transformation of elasticity, plasticity, and viscosity during the hydration of cement [26]. Therefore, three representative parameters of rheology, plastic viscosity, thixotropy and yield stress, will be investigated and correlated to the mechanical properties of 3DP SAC paste. Finally, controllable setting time, yield stress, and plastic viscosity will be discussed and summarized.

## 2. Materials and Methods

### 2.1. Raw Materials

**Figure 1:**
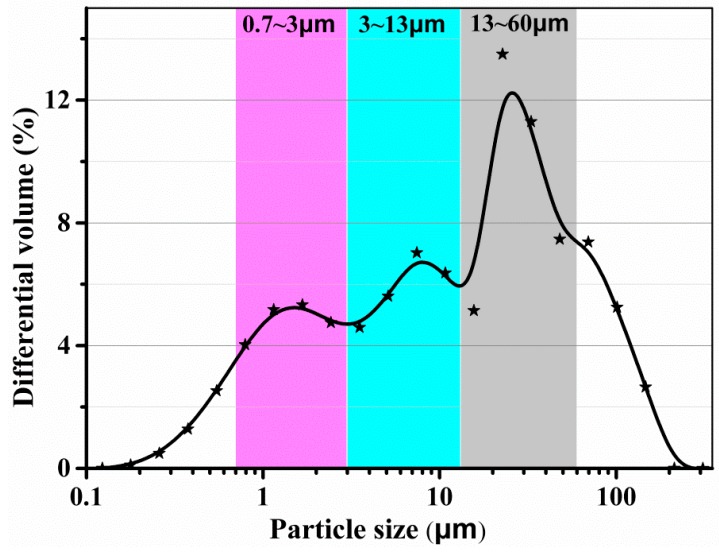
Particle size distribution of the SAC.

SAC (42.5 grade, Zhonglian Company, Qufu, China) was used as the 3D printing cementitious materials in this paper. The chemical components and particle size distribution of the SAC are shown in Table 1 and Figure 1, respectively. Hydroxypropyl methyl cellulose (HPMC, 75000 mPa·s, Heda Company, Zibo, China) was used as a viscosity modifier to increase the viscosity of the 3DP SAC paste. Water reducing agent (WRA, polycarboxylate, Shandong Acadamy of Building Research, Jinan, China) with a water reducing rate of 32% was used to improve the printability of 3DP SAC paste. TA was introduced to adjust the setting time, rheological properties and control the structure of the 3DP SAC paste.

### 2.2. Fabrication Procedures

The detailed mix proportion of raw materials for preparing the 3DP SAC paste is listed in Table 2 and the fabrication procedures are as follows:

(1) SAC was mechanically blended with HPMC and TA at the speed of 32 rpm for 30 min to get a uniform solid mixture by a V-shape blender (VH01, Jinhong General Machinery Co. Ltd., Xianyang, China);

(2) The above mixture was then mixed with water at the speed of 285 rpm for 3 min in a cement paste mixer (NJ-160, Jianyi Instrument and Machinery Co. Ltd., Wuxi, China), and WRA was dropwise added into the fresh paste;

(3) The fresh paste was put into a 3D printer (Pottery artist, Dianfeng Company, Yueyang, China) and then extruded from a nozzle with the size of 3.0 mm based on the preset models;

(4) Printed samples were cured in a curing chamber with the relative temperature of 20 °C and humidity of 95% for 24 h.

### 2.3. Testing Methods

#### 2.3.1. Particle Size Distribution

Particle size distribution of the SAC was measured by a particle size analyzer (BECKMAN COULTER’s LS13320, Beckman Coulter Inc., Pasadena, CA, USA).

#### 2.3.2. 3DP System

**Figure 2:**
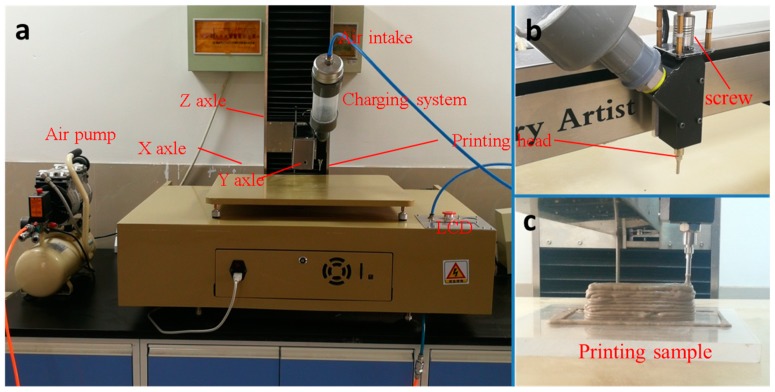
(**a**) 3D printer, (**b**) extrusion system and (**c**) printed sample.

The 3DP system consists of extrusion system, liquid crystal display (LCD) operation panel, air pump, XYZ axle and charging system, and the macrograph of 3D printer is shown in Figure 2a,b. The largest printing size of 3D printer is 0.8 m × 0.8 m × 0.8 m. The SAC paste was continuously extruded through the manner of screw mixing under the air pump pressure of 0.3 MPa, loading speed of 15 mm/s and printing speed of 10 mm/s. Figure 2c shows a printed sample with the size of 25 mm × 25 mm × 70 mm (10 layers).

#### 2.3.3. Setting Time

The Vicat apparatus was used to measure the initial (penetration depth of 36 ± 1 mm) and final setting time (penetration depth of 0 mm) of 3DP SAC paste according to the GB-T 1346-2011 [27].

#### 2.3.4. Ultimate Deformation Rate

Ultimate deformation rate was calculated as per the positive strain, which indicates the largest deformation of samples after printing. In this paper, the ultimate deformation rate of a sample is expressed by Equation (1):

(1) $$D=\frac{\left[\left(l-l_{0}\right)+\left(w-w_{0}\right)+\left(h_{0}-h\right)\right]}{3\left(l_{0}+w_{0}+h_{0}\right)}\times 100\%$$

where D is the ultimate deformation rate, *l*_0_, *w*_0_, and *h*_0_ represent the size of model (designed size); *l*, *w* and *h* represent the stable size of samples after printing.

#### 2.3.5. Hydration Heat Evolution

The heat flow and cumulative heat of SAC paste with the different addition of TA were continuously collected for 24 h by a conduction calorimeter (TAM Air C80, Thermometric Company, Stockholm, Sweden).

#### 2.3.6. Rheological Properties

**Figure 3:**
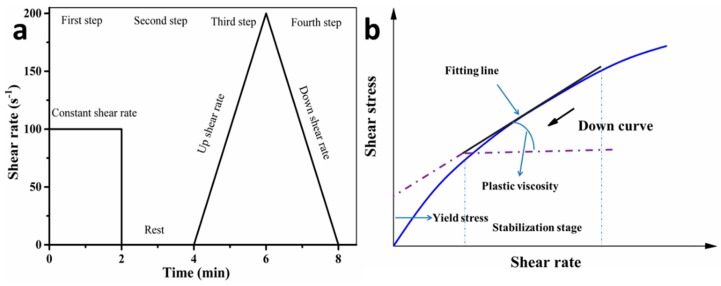
(**a**) The test program of rheology and (**b**) The fitting line of plastic viscosity and yield stress was obtained by the calculation of Bingham model based on the down curve of shear stress

The rheological properties of SAC paste, including shear rate, shear stress and apparent viscosity, were measured by a rotational rheometer (Kinexus Lab+, Malvern, UK). The four-step test procedure of rheology measurement is shown in Figure 3a. Firstly, the fresh SAC paste was measured under a constant shear rate of 100 s^−1^ in the first 2 min, followed by 2 min of rest. Then, 3D printing paste was measured with an increasing shear rate from 0 to 200 s^−1^ in 4–6 min and a descending shear rate in 6 –8 min. The rheological properties of fresh SAC paste are related to the viscosity, elasticity, and plasticity, and the corresponding rheological curve is similar to Bingham fluid. Besides, Bingham model was obtained based on the ideal Saint-Venant’s plasticity and Newton’s viscosity law. It can well reflect the viscosity and plasticity of SAC paste. Therefore, plastic viscosity and yield stress was obtained by the calculation of Bingham model in Figure 3b.

Thixotropy is a time-dependent shear thinning property, which can reflect the viscosity development of testing samples with the variation of shear force. Since the viscosity of fresh 3DP SAC paste has a great change before and after the extrusion from nozzle, thixotropy should be well optimized to ensure the printed samples to keep its shape without collapse.

Under a constant shear rate, shear stress of fresh SAC paste gradually increases until it reaches the maximum value, and then decreases to a steady value. This phenomenon is caused by the breakdown of 3D structures, which affects the thixotropy significantly. Qian et al. [28] proposed the thixotropic index to explain the variation of thixotropy. This thixotropic index depicts the relationship between static and dynamic yield stress. The larger the thixotropy index, the better the thixotropy. It can be expressed by Equation (2):

(2) $$I_{thix}=\tau_{i}/\tau_{e}$$

where *I_thix_*, *τ_i_* and *τ_e_* are the thixotropic index, maximum value and steady value.

Under a varying shear rate, shear stress of fresh SAC paste is positive correlated the shear rate. The hysteresis loop surrounded by the up and down curves is related to the breakdown of 3D structures and thixotropy of SAC paste. The area of hysteresis loop can be expressed by Equation (3):

(3) $$A=\int_{\gamma min}^{\gamma max}\eta_{1}\gamma d\gamma -\int_{\gamma min}^{\gamma max}\eta_{2}\gamma d\gamma$$

where A is the area of hysteresis loop, *γ* is the shear rate, *η*_1_ and *η*_2_ represent the up and down curves.

#### 2.3.7. Mechanical Strength

The mechanical properties of 3DP SAC paste, including compressive strength and flexural strength, were tested by a universal testing machine (Criterion 40, MTS Systems Corporation, Eden Prairie, MN, USA) with the capacity of 300 kN. For the compressive strength measurement, six cubic samples with the dimensions of 20 mm × 20 mm × 20 mm were tested at the loading rate of 0.3 kN/s. In addition, three-point bending test on three prism specimens in dimensions of 20 mm × 20 mm × 60 mm was conducted as per the procedure prescribed by ASTM C 651-15.

## 3. Results and Discussion

### 3.1. Setting Time and Hydration Evolution

**Figure 4:**
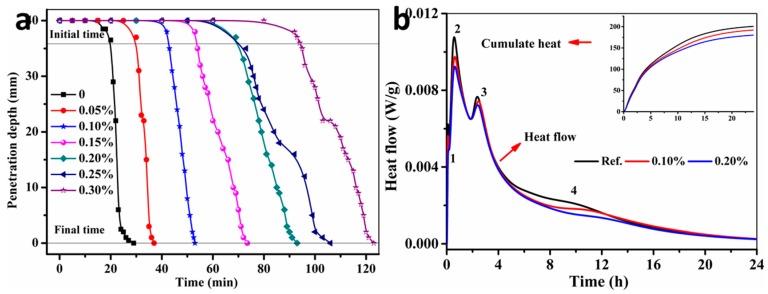
(**a**) The setting time and (**b**) hydration heat of the SAC paste with different additions of tartaric acid (TA).

The setting time and hydration evolution are of great importance to control the printing time and quality of the SAC. In this paper, TA was used as the retarder to optimize both parameters to satisfy the requirements of 3DP. Figure 4 shows that the setting time and hydration evolution of fresh SAC paste with different additions of TA. From Figure 4a, it is clear to see that the addition of TA presents a significant retardation for the fresh SAC paste. Compared with the controlled SAC paste without TA, the initial and final setting time of SAC paste can be prolonged to 22–98 min and 30–123 min by adding 0.05% to 0.30% of TA, respectively. In addition, Figure 4b shows the heat flow and cumulative heat curves of SAC paste with different additions of TA, which can be divided into four stages. The first stage is the dissolution of solid powders to water. The second and third stage result from the formation of AFt (C_3_A·3C$·H_32_; C = CaO, A = Al_2_O_3_, $ = SO_3_, H = H_2_O) which is the major hydration product of C_3_A and C_4_A_3_$ (C_3_A + 3C$ + 32H → C_3_A·3C$·H_32_; C_4_A_3_$ + 2C$ + 38H → C_3_A·3C$·H_32_ + 2AH_3_). The fourth stage is caused by the formation of AFm (C_3_A·C$·H_12_; C_4_A_3_$ + 18H → C_3_A·C$·H_12_ + 2AH_3_) which generates from the hydration of C_4_A_3_$ in the lack of gypsum [29]. More importantly, it also indicates that the addition of TA significantly inhibits the transformation from AFt to AFm in the SAC. The possible reason is that a water film can be generated on the surface of C_3_A and C_4_A_3_$ due to the high affinity (–OH groups) of TA to Al^3+^ and thus reduce the hydration of both minerals. In addition, TA, as a kind of calcium chelating ligand, can coordinate with Ca^2+^ on the surface of cement particles by the function of COO^−^ groups, which can generate a complex only sparingly soluble in water [30]. Based on the two reasons above, the hydration rate and cumulate heat of SAC paste can be reduced with the increasing addition of TA.

### 3.2. Rheological Properties

Rheological properties of 3DP SAC paste are very crucial to guarantee the build-up of structure with a low deformation rate. The apparent viscosity is the sum of internal friction among the solid particles, and shear stress is an indicator to resist the external forces from two shear plane. The relationship can be expressed by Equation (4):

(4) τ=ηγ

where *τ* represents the shear stress; *η* is the apparent viscosity; *γ* is the shear rate. It should be noted that this equation is only valid for Bingham fluid with zero yield stress.

**Figure 5:**
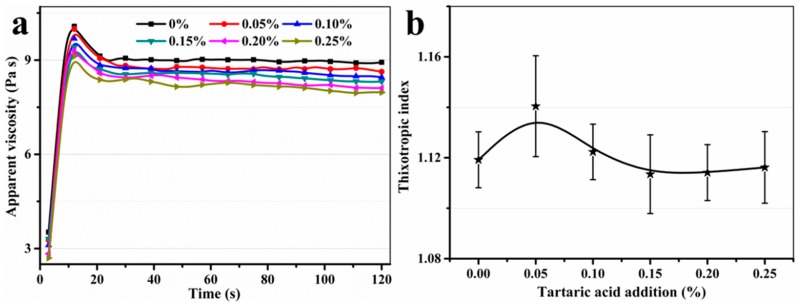
(**a**) The apparent viscosity and (**b**) thixotropic index of fresh SAC paste with different additions of TA under a constant shear rate of 100 s^−1^.

Figure 5 presents the apparent viscosity and thixotropic index of fresh SAC paste with different additions of TA under a constant shear rate of 100 s^−1^. From Figure 5a, it indicates that apparent viscosity of fresh SAC paste decreases with the increasing addition of TA until it reaches to an equilibrium value. However, the thixotropic index (average value of three times in this paper) of fresh SAC paste increases briefly and then decreases again, as shown in Figure 5b. A better thixotropy of fresh SAC paste can be achieved when addition of TA is 0.05%. This phenomenon can be explained by that the water film formed on the surface of cement particles can be damaged under a higher shear rate when the addition of TA is relatively low (0.05%). In this case, the shear stress only changes little. With the increase of stirring time, the fresh SAC paste becomes more uniform, and water film will easily form again on the surface of cement particles, which causes the descending shear stress. However, the water film is very stable and difficult to be damaged when the addition of TA is higher than 0.05%.

**Figure 6:**
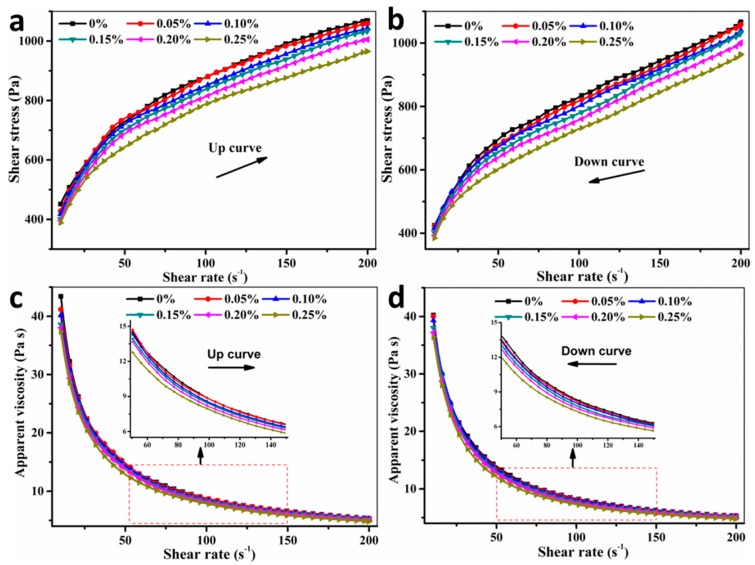
The rheology properties of the fresh SAC paste with different additions of TA under a varying shear rate from 10–200 s^−1^: (**a**,**b**) shear stress of up curves and down curves; (**c**,**d**) apparent viscosity of up curves and down curves.

Figure 6 shows the apparent viscosity and shear stress of fresh SAC paste with different additions of TA under a varying shear rate from 10–200 s^−1^. It can be seen that the shear stress and apparent viscosity gradually decrease with the increasing addition of TA. More specifically, the fresh SAC paste exists a shear-thinning behavior, which belongs to the pseudoplastic fluid. The reason is that the internal particles are likely to slide under the high shear rate, which makes the internal fraction decrease and improves the dispersion of cement particles.

**Figure 7:**
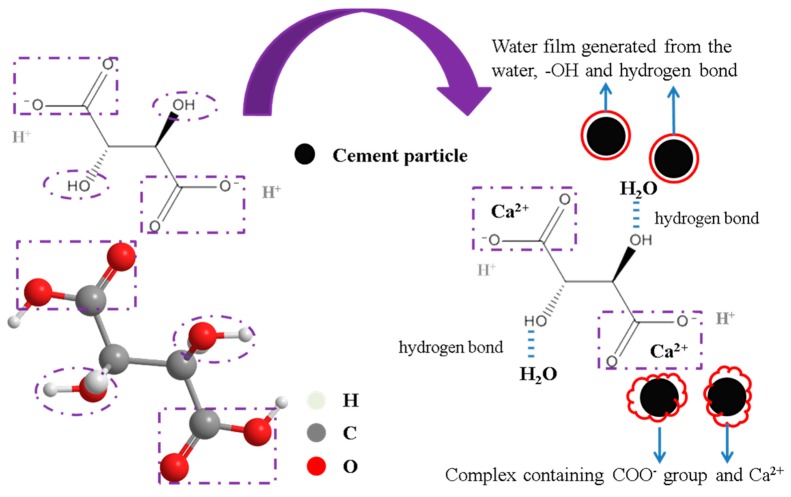
The retardation and rheology mechanism of TA to the 3D printing sulphoaluminate cement (3DP SAC) paste.

Figure 7 shows the retardation and rheology mechanism of TA in the 3DP SAC paste. On the one hand, –OH groups from the TA can induce the formation of water film on the surface of cement particles through the hydrogen bonds. This behavior can facilitate the relative motion among different particles and decrease the internal friction. On the other hand, COO^−^ groups from the TA can coordinate with Ca^2+^ released from the hydration of C_4_A_3_$ to generate a stable complex which prevents the water contacting with cement particles. Therefore, more free water exists in the fresh SAC paste, which is beneficial to reduce the viscosity of the mixture [31,32]. By the two ways above, the hydration and rheology properties of 3DP SAC paste are significantly influenced by adding TA.

The plastic viscosity presents the difficulty level of structural failure when the fresh SAC paste is at rest, and the yield stress is the minimum force to actuate the paste to flow [33]. The relationship of shear rate, yield stress and plastic viscosity can be expressed by Equation (5):

(5) $$\tau =\tau_{0}+\mu \gamma$$

where *τ* and *τ*_0_ represent the shear stress and yield stress, respectively; *μ* is the plastic viscosity; *γ* is the shear rate.

**Figure 8:**
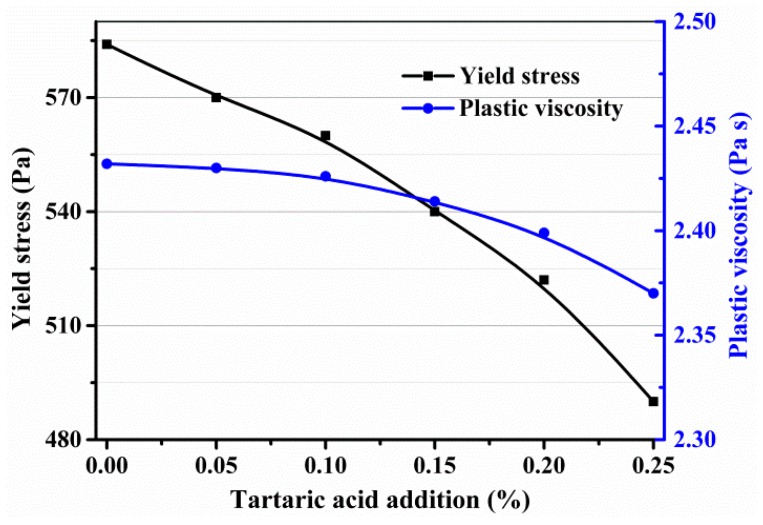
The plastic viscosity and yield stress of the fresh SAC paste with different additions of TA based on the Bingham model.

Based on the fitting curves, the plastic viscosity and yield stress of fresh SAC paste with different additions of TA are drawn in Figure 8. It can be seen that, with the increasing addition of TA, the plastic viscosity and yield stress gradually decrease from 584 to 490 Pa and 2.432 to 2.370 Pa·s, respectively.

### 3.3. Thixotropy Property

**Figure 9:**
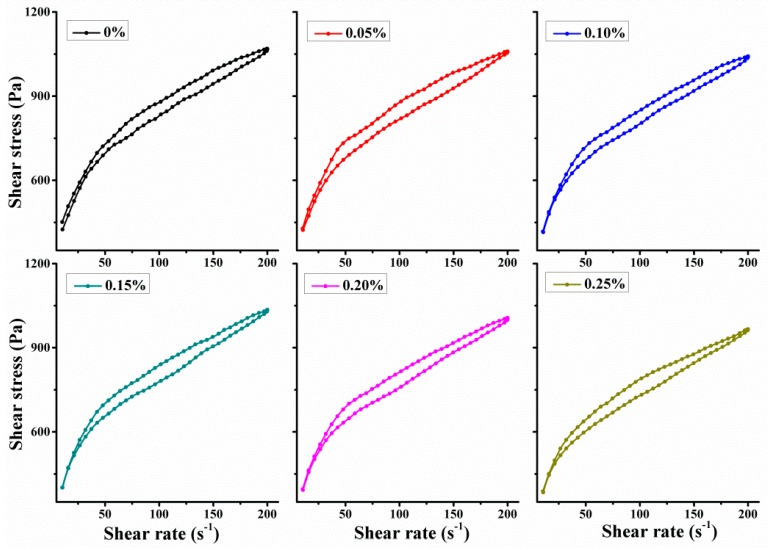
The hysteresis loop surrounded by up and down curves of shear stress.

3DP technology requires the cementitious materials to present a high viscosity and low deformation. Therefore, a good thixotropy should ensure the build-up of 3D structures. Figure 9 shows the hysteresis loop curves of up and down shear stress. The area of the hysteresis loop indicates the thixotropy variation, which can be expressed as:

(6) $$A=\int_{10}^{200}C_{1}d\gamma -\int_{10}^{200}C_{2}d\gamma$$

where A is the area of hysteresis loop, *γ* is the shear rate, *C*_1_ and *C*_2_ represent the up and down curves.

**Figure 10:**
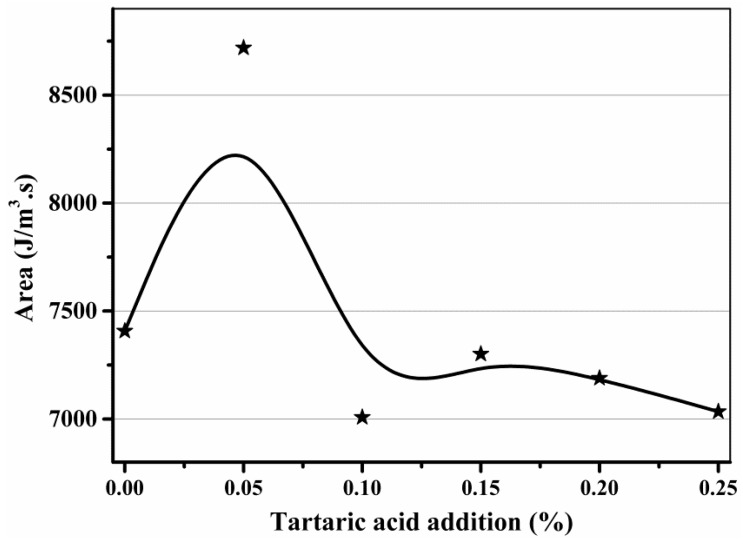
Thixotropy evaluation of the fresh SAC paste with different additions of TA from 0–0.25%.

Thixotropy variation of 3DP SAC paste with different additions of TA is shown in Figure 10. It indicates that the effectiveness of thixotropy sharply increases and then decreases until it reaches to a steady value. The largest area of hysteresis loop appears when the addition of TA is at 0.05%. This result is similar to the thixotropic index under a constant shear rate. The reason is that the water film formed on the surface of cement particles will be damaged under a larger shear rate. At this time, the addition of tartaric acid is relatively small (0.05%) and the shear stress decreases slightly. As the shear rate decreasing, the water film gradually recovers and the shear stress decreases significantly. These two aspects lead to the increase of thixotropy area. Besides, water film will be stable and difficult to damage when the addition of tartaric acid is relatively large.

### 3.4. Structure Build-Up and Mechanical Strength

**Figure 11:**
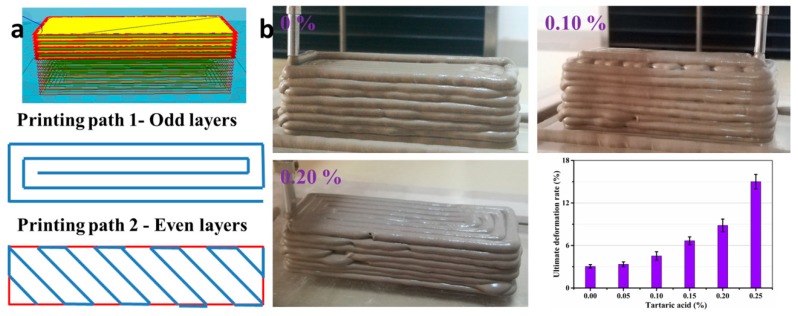
(**a**) The designed model and printing path and (**b**) the macrograph and ultimate deformation rate of the fresh SAC paste for different additions of TA.

3DP technology requires the cementitious materials to present good stacking character and shape stability after extrusion from 3D printer. Therefore, a low deformation rate of printed samples should be guaranteed, which is targeted less than 10% in this paper. Figure 11 shows the designed model, printing path, macrograph, and the ultimate deformation rate of 3DP SAC paste with different additions of TA. It indicates that the ultimate deformation rate gradually increases with the increasing addition of TA. Meanwhile, an excellent structure of 3DP SAC paste can be fabricated (ultimate deformation rate <10%) by 3DP technique when the addition of TA is lower than 0.20%. Based on the above results, the addition of TA should be controlled within the range of 0–0.20% to ensure the good printability and proper rheological properties of 3DP SAC paste. At this time, plastic viscosity and yield stress should be controlled within the range of 2.432–2.399 Pa·s and 584–522 Pa, respectively. Besides, the initial setting time can be controlled within the range of 22–69 min.

**Figure 12:**
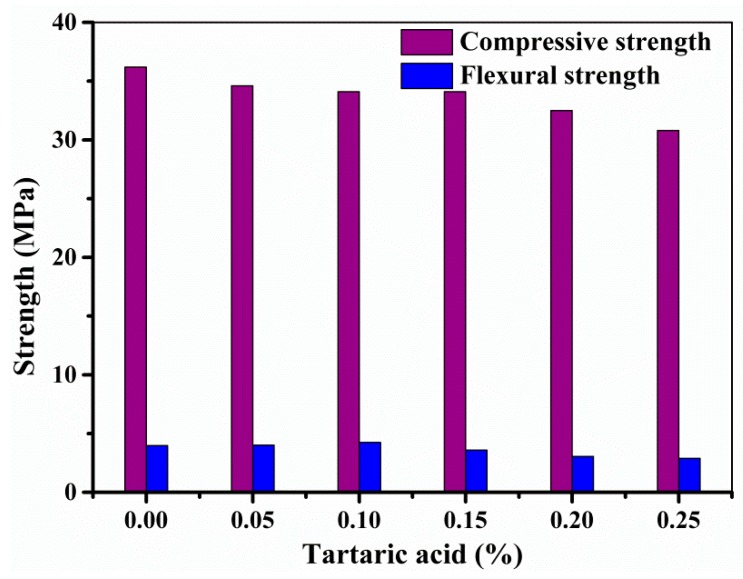
The compressive and flexural strength of the SAC with different additions of tartaric acid after curing 1 day.

Retardants usually have no effect or even slight improvement on the long-term strength of cement-based materials while the reverse phenomenon is true for the early strength. Figure 12 presents the compressive and flexural strength of 3DP SAC paste with different additions of TA after curing 1 day. It can be found that the addition of TA has little influence on the mechanical strength of 3DP SAC paste when the addition of TA increases from 0 to 0.25%.

## 4. Conclusions

In this study, SAC paste was fabricated by extrusion-based 3DP technique. To achieve a good printability, TA was used as a retardant to adjust the setting time to satisfy the printing requirements, and the effect of TA on the hydration evolution, rheological properties and mechanical strength was also investigated. The experimental results indicated that a moderate addition of TA into SAC enables it is available to develop 3DP SAC paste with a low deformation rate, controllable rheological properties and satisfied mechanical strength. The main conclusions of this study are highlighted as below:

1. The initial and final setting time of the SAC paste can be controlled within the range of 22–98 min and 30–123 min, respectively. With the TA addition of 0–0.30%, the setting time of SAC paste can achieve the required printing time.
2. With the increase of TA addition from 0 to 0.25%, the plastic viscosity and yield stress gradually decrease from 584 to 490 Pa and 2.432 to 2.370 Pa·s, respectively. Meanwhile, it can also slightly improve the thixotropy.
3. With the initial setting is within the range of 22–69 min, an excellent 3D structure of the printed SAC paste with the ultimate deformation rate less than 10% can be manufactured.
4. The compressive and flexural strength of the 3D printed SAC remains unchanged when the addition of TA increases from 0 to 0.25%.

## Figures and Tables

**Table 1:** The chemical components of the sulphoaluminate cement (SAC) (% by mass).

CaO	Al_2_O_3_	SO_3_	SiO_2_	Fe_2_O_3_	TiO_2_	K_2_O	MgO	Others	Loss on Ignition
49.50	20.17	14.91	8.51	1.97	1.57	0.90	0.77	0.73	0.97

**Table 2:** The detailed mix proportion of raw materials (%).

Raw Materials	SAC	WRA	HPMC	Tartaric Acid	Water
Mix proportion	100	0.30	0.40	0–0.25	35

## References

[1] Ligon S.C., Liska R., Stampfl J., Gurr M., Mulhaupt R. (2017). Polymers for 3D Printing and Customized Additive Manufacturing. Chem. Rev..

[2] Siqueira G., Kokkinis D., Libanori R., Hausmann M.K., Gladman A.S., Neels A., Tingaut P., Zimmermann T., Lewis J.A., Studart A.R. (2017). Cellulose Nanocrystal Inks for 3D Printing of Textured Cellular Architectures. Adv. Funct. Mater..

[3] Chen Q., Mangadlao J.D., Wallat J., De Leon A., Pokorski J.K., Advincula R.C. (2017). 3D Printing Biocompatible Polyurethane/Poly(lactic acid)/Graphene Oxide Nanocomposites: Anisotropic Properties. ACS Appl. Mater. Interfaces.

[4] Shie M.Y., Chang W.C., Wei L.J., Huang Y.H., Chen C.H., Shih C.T., Chen Y.W., Shen Y.F. (2017). 3D Printing of Cytocompatible Water-Based Light-Cured Polyurethane with Hyaluronic Acid for Cartilage Tissue Engineering Applications. Materials.

[5] Fu K., Wang Y., Yan C., Yao Y., Chen Y., Dai J., Lacey S., Wang Y., Wan J., Li T. (2016). Graphene Oxide-Based Electrode Inks for 3D-Printed Lithium-Ion Batteries. Adv. Mater..

[6] Pyo S.H., Wang P., Hwang H.H., Zhu W., Warner J., Chen S. (2017). Continuous Optical 3D Printing of Green Aliphatic Polyurethanes. ACS Appl. Mater. Interfaces.

[7] Xu J., Ding L., Love P.E.D. (2017). Digital reproduction of historical building ornamental components: From 3D scanning to 3D printing. Autom. Constr..

[8] Duballet R., Baverel O., Dirrenberger J. (2017). Classification of building systems for concrete 3D printing. Autom. Constr..

[9] Wolfs R.J.M., Bos F.P., Salet T.A.M. (2018). Correlation between destructive compression tests and non-destructive ultrasonic measurements on early age 3D printed concrete. Constr. Build. Mater..

[10] Khalil N., Aouad G., El Cheikh K., Rémond S. (2017). Use of calcium sulfoaluminate cements for setting control of 3D-printing mortars. Constr. Build. Mater..

[11] Le T.T., Austin S.A., Lim S., Buswell R.A., Gibb A.G.F., Thorpe T. (2012). Mix design and fresh properties for high-performance printing concrete. Mater. Struct..

[12] Le T.T., Austin S.A., Lim S., Buswell R.A., Law R., Gibb A.G.F., Thorpe T. (2012). Hardened properties of high-performance printing concrete. Cem. Concr. Res..

[13] Sanjayan J.G., Nematollahi B., Xia M., Marchment T. (2018). Effect of surface moisture on inter-layer strength of 3D printed concrete. Constr. Build. Mater..

[14] Wolfs R.J.M., Bos F.P., Salet T.A.M. (2018). Early age mechanical behaviour of 3D printed concrete: Numerical modelling and experimental testing. Cem. Concr. Res..

[15] Shakor P., Sanjayan J., Nazari A., Nejadi S. (2017). Modified 3D printed powder to cement-based material and mechanical properties of cement scaffold used in 3D printing. Constr. Build. Mater..

[16] Ma G., Li Z., Wang L. (2018). Printable properties of cementitious material containing copper tailings for extrusion based 3D printing. Constr. Build. Mater..

[17] Ketel S., Falzone G., Wang B., Washburn N., Sant G. (2018). A printability index for linking slurry rheology to the geometrical attributes of 3D-printed components. Cem. Concr. Compos..

[18] Soltan D.G., Li V.C. (2018). A self-reinforced cementitious composite for building-scale 3D printing. Cem. Concr. Compos..

[19] Buswell R.A., Silva W.R.L.D., Jones S.Z., Dirrenberger J. (2018). 3D printing using concrete extrusion: A roadmap for research. Cem. Concr. Res..

[20] Reiter L., Wangler T., Roussel N., Flatt R.J. (2018). The role of early age structural build-up in digital fabrication with concrete. Cem. Concr. Res..

[21] Huang Y., Qian J., Liang J., Liu N., Li F., Shen Y. (2016). Characterization and calorimetric study of early age hydration behaviors of synthetic ye’elimite doped with the impurities in phosphogypsum. J. Therm. Anal. Calorim..

[22] Marchon D., Kawashima S., Bessaies-Bey H., Mantellato S., Ng S. (2018). Hydration and rheology control of concrete for digital fabrication: Potential admixtures and cement chemistry. Cem. Concr. Res..

[23] Ma B., Peng Y., Tan H., Jian S., Zhi Z., Guo Y., Qi H., Zhang T., He X. (2018). Effect of hydroxypropyl-methyl cellulose ether on rheology of cement paste plasticized by polycarboxylate superplasticizer. Constr. Build. Mater..

[24] Tan H., Zou F., Ma B., Guo Y., Li X., Mei J. (2017). Effect of competitive adsorption between sodium gluconate and polycarboxylate superplasticizer on rheology of cement paste. Constr. Build. Mater..

[25] Wang Q., Cui X., Wang J., Li S., Lv C., Dong Y. (2017). Effect of fly ash on rheological properties of graphene oxide cement paste. Constr. Build. Mater..

[26] Roussel N. (2018). Rheological requirements for printable concretes. Cem. Concr. Res..

[27] GB-T 1346-2011 (2011). Test Methods for Water Requirement of Normal Consistency, Setting Time and Soundness of the Cement.

[28] Qian Y., Lesage K., El Cheikh K., De Schutter G. (2018). Effect of polycarboxylate ether superplasticizer (PCE) on dynamic yield stress, thixotropy and flocculation state of fresh cement pastes in consideration of the Critical Micelle Concentration (CMC). Cem. Concr. Res..

[29] Li L., Zhou X., Li Y., Gong C., Lu L., Fu X., Tao W. (2017). Water absorption and water/fertilizer retention performance of vermiculite modified sulphoaluminate cementitious materials. Constr. Build. Mater..

[30] Zajac M., Skocek J., Bullerjahn F., Haha M.B. (2016). Effect of retarders on the early hydration of calcium-sulpho-aluminate (CSA) type cements. Cem. Concr. Res..

[31] Lu Z., Hanif A., Sun G., Liang R., Parthasarathy P., Li Z. (2018). Highly dispersed graphene oxide electrodeposited carbon fiber reinforced cement-based materials with enhanced mechanical properties. Cem. Concr. Compos..

[32] Lu Z., Hanif A., Ning C., Shao H., Yin R., Li Z. (2017). Steric stabilization of graphene oxide in alkaline cementitious solutions: Mechanical enhancement of cement composite. Mater. Des..

[33] Lu Z., Hanif A., Lu C., Sun G., Cheng Y., Li Z. (2018). Thermal, mechanical, and surface properties of polyvinyl alcohol (PVA) polymer modified cementitious composites for sustainable development. J. Appl. Polym. Sci..

